# Signs of Hemolysis Predict Mortality and Ventilator Associated Pneumonia in Severe Acute Respiratory Distress Syndrome Patients Undergoing Veno-Venous Extracorporeal Membrane Oxygenation

**DOI:** 10.1097/MAT.0000000000002278

**Published:** 2024-07-30

**Authors:** Emanuele Rezoagli, Michela Bombino, Lorraine B. Ware, Eleonora Carlesso, Roberto Rona, Giacomo Grasselli, Antonio Pesenti, Giacomo Bellani, Giuseppe Foti

**Affiliations:** From the * School of Medicine and Surgery, University of Milan-Bicocca, Monza, Italy; † Department of Emergency and Intensive Care, Fondazione Istituto di Ricovero e Cura a Carattere Scientifico (IRCCS) San Gerardo dei Tintori, Monza, Italy; ‡ Department of Medicine, Allergy, Pulmonary, and Critical Care Medicine, Vanderbilt University Medical Center, Nashville, Tennessee; § Department of Pathology, Microbiology and Immunology, Vanderbilt University Medical Center, Nashville, Tennessee; ¶ Department of Medical Physiopathology and Transplants, University of Milan, Milano, Italy; ∥ Department of Anesthesia, Critical Care and Emergency, Fondazione Istituto di Ricovero e Cura a Carattere Scientifico (IRCCS) Ca’ Granda—Ospedale Maggiore Policlinico, Milan, Italy; # Centre for Medical Sciences—CISMed, University of Trento, Trento, Italy; ** Department of Anesthesia and Intensive Care, Santa Chiara Hospital, Trento, Italy.

**Keywords:** COHb, haptoglobin, ARDS, ECMO, mortality

## Abstract

Cell-free hemoglobin (CFH) is used to detect hemolysis and was recently suggested to trigger acute lung injury. However, its role has not been elucidated in severe acute respiratory distress syndrome (ARDS) patients undergoing extracorporeal membrane oxygenation (ECMO). We investigated the association of carboxyhemoglobin (COHb) and haptoglobin—two indirect markers of hemolysis—with mortality in critically ill patients undergoing veno-venous ECMO (VV-ECMO) with adjusted and longitudinal models (primary aim). Secondary aims included assessment of association between COHb and haptoglobin with the development of ventilator-associated pneumonia (VAP) and with hemodynamics. We retrospectively collected physiological, laboratory biomarkers, and outcome data in 147 patients undergoing VV-ECMO for severe ARDS. Forty-seven patients (32%) died in the intensive care unit (ICU). Average levels of COHb and haptoglobin were higher and lower, respectively, in patients who died. Higher haptoglobin was associated with lower pulmonary (PVR) and systemic vascular resistance, whereas higher COHb was associated with higher PVR. Carboxyhemoglobin was an independent predictor of VAP. Both haptoglobin and COHb independently predicted ICU mortality. In summary, indirect signs of hemolysis including COHb and haptoglobin are associated with modulation of vascular tone, VAP, and ICU mortality in respiratory ECMO. These findings suggest that CFH may be a mechanism of injury in this patient population.

## Background

Extracorporeal membrane oxygenation (ECMO) is reserved for the most severe cases of acute respiratory distress syndrome (ARDS) including patients with refractory hypoxemia^1,2^ or when lung protective ventilation is not possible.^3^ Although overt hemolysis is infrequent in ECMO, subclinical hemolysis is common but not considered clinically relevant.^4^ Recently, the significance of hemolysis in ECMO has been increasingly recognized. In cardiopulmonary bypass, hemolysis has been linked to the development of acute kidney injury,^5–7^ and haptoglobin supplementation seems to decrease its incidence.^8^ Overt hemolysis during ECMO was associated with increased mortality and organ failures.^9^

When stored in red blood cells, hemoglobin transports oxygen and is essential for human life. On the other hand, when outside red blood cells, cell-free hemoglobin (CFH), is a powerful activator of oxidative stress leading to organ damage.^10^ Cell-free hemoglobin toxicity has several mechanisms: vasoconstriction by nitric oxide scavenging; activation of neutrophils; and generation of oxidative stress leading to inflammation and vascular permeability.^11^ Cell-free hemoglobin and heme increase inflammation and permeability in acute lung injury and ARDS by damaging both endothelial and alveolar epithelial barriers.^12^ Endogenous scavengers of CFH and free heme (haptoglobin and hemopexin) help to maintain normal redox balance in the extracellular compartment and organ tissues and are proposed as therapeutic agents to counteract the effects of CFH.^13^

Cell-free hemoglobin is the main protein detected in plasma to quantify—directly—the degree of intravascular hemolysis. However, the measurement of free hemoglobin requires laboratory assays that are not always routinely available in clinical practice.^13^ Very recently, carboxyhemoglobin (COHb) has been proposed as a reliable diagnostic marker of hemolysis in critically ill patients.^14,15^ Carboxyhemoglobin is formed when CFH is metabolized by heme-oxygenase 1 producing cardiac output (CO) which can react with CFH leading to COHb. Hariri *et al.*^15^ reported that COHb was an accurate diagnostic biomarker of intravascular hemolysis with an area under the curve (AUC) of 93%. Haptoglobin is another indirect biomarker of hemolysis because of its neutralizing activity against CFH.^16^ Haptoglobin is negatively correlated with CFH^17^ and in pediatric patients undergoing cardiac surgery lower levels are associated with mortality, nosocomial infection, and inflammatory biomarkers.^18^

The aim of the current study is to analyze the association of indirect measures of hemolysis—such as plasma levels of haptoglobin and COHb—with intensive care unit (ICU) mortality (primary aim) and other important outcomes (*eg*, incidence of secondary infections such as ventilator-associated pneumonia [VAP], and pulmonary and systemic vascular resistance—secondary aims) in severe ARDS patients supported with VV-ECMO. We used these indirect signs of hemolysis, as opposed to a direct measurement of CFH, because this latter measure was not routinely available at our institution.

## Materials and Methods

### Patient Population

This is a retrospective analysis of a large cohort of patients with severe ARDS treated with VV-ECMO support, between October 2003 and May 2019 in our ICU, which has been a referral center for this condition since 1988.

We included in this study the patients fulfilling the following inclusion criteria:

1. Use of a magnetic levitation centrifugal pump.
2. Age ≥16.
3. Acute respiratory distress syndrome diagnosis according to the Berlin criteria.
4. Daily available levels of haptoglobin and COHb during VV-ECMO.

The study was carried out in accordance with the principles laid down in the 2013 revision of the Declaration of Helsinki. The local Ethics Committee of San Gerardo Hospital approved the study, and the informed consent was waived due to the retrospective study design.

### Extracorporeal Membrane Oxygenation Circuit and Management

A complete description of ECMO management and weaning process has been described elsewhere.^19^

The ECMO circuit was similar in all patients, including a permanent life support (PLS) system or a CARDIOHELP system by Maquet (Rastatt, Germany), a drainage cannula (HLS Multistage, Maquet), and a return cannula (Bio-Medicus, Medtronic, Minneapolis).

Extracorporeal Membrane Oxygenation circuits were mainly changed for two reasons: signs of clotting (mainly indicated by a gradual increase in D-dimers associated with platelets and fibrinogen consumption), or membrane lung (ML) failure (increase in pressure drop across the ML with concurrent decrease in ECMO blood flow). Per our practice, all patients had a pulmonary artery catheter inserted before or right after ECMO initiation for central hemodynamic monitoring and mixed venous blood gas collections.

### Data Collection

Anthropometric data (height, weight, age, body mass index [BMI]), etiology of ARDS, basal laboratory and blood gas analysis data, and ventilator settings were gathered before ECMO initiation. We calculated the following outcome predictive scores: Sequential Organ Failure Assessment (SOFA),^20^ Respiratory Extracorporeal Membrane Oxygenation Survival Prediction (RESP),^21^ and PRedicting dEath for SEvere ARDS on VV-ECMO (PRESERVE).^22^

Every day during the ECMO run we gathered the following data:

1. Hemodynamic parameters: heart rate (HR), systemic blood pressure, central venous pressure, pulmonary artery pressure, pulmonary artery occlusion pressure (PAOP), and CO. The use of vasopressors and dosage were also collected. Calculation of pulmonary (PVR) and systemic vascular resistances (SVRs) were computed according to standard formulae.
2. Mechanical ventilation settings: respiratory rate, tidal volume, peak and mean airway pressure, positive end expiratory pressure (PEEP), inspired oxygen fraction (FiO_2_).
3. Blood gas analysis: arterial, mixed venous, before and after the ML were gathered once a day at FiO_2_ 1.0. The shunt fraction was calculated according to the Berggren equation.
4. Daily laboratory data were collected from medical records: Hb, D-dimer, and creatinine. We routinely monitor hemolysis with the consumption of haptoglobin, lactate dehydrogenase (LDH) measurements and follow the trend in COHb on blood gases. All patients had one determination of iron metabolism (iron, ferritin, and transferrin), C-reactive protein and fibrinogen at ICU admission.
5. Extracorporeal membrane oxygenation parameters and performance: extracorporeal blood flow, sweep gas flow, pressures across the artificial lung, and negative inlet pressure were collected.
6. Red blood cell component transfusion requirements (RBC) need for ECMO circuit change and therefore number of circuit used during the ECMO run, bloodstream infections and incidence of VAP, and need for continuous renal replacement therapy (CRRT) were also collected. Ventilator-associated pneumonia was defined as the presence of pulmonary infiltrate on the chest radiograph at least 48 hours after intubation together with the presence of positive quantitative bacterial culture in bronchoalveolar lavage or in distal bronchoaspirate using a closed-loop aspiration system.

### Statistical Analysis

The normality of data distribution was tested using the Shapiro–Wilk test. Continuous variables were expressed as mean ± standard deviation (SD) or as median (interquartile range) as appropriate. Categorical data were described as proportion (percentage). Patients were stratified into survivors and nonsurvivors. Differences between continuous data in survivors and nonsurvivors were tested using unpaired Student’s t-test or Wilcoxon rank-sum test, as appropriate. Differences in categorical data were tested using Chi-square or Fisher’s exact test. We performed univariate linear regression analysis to investigate variables associated with indirect signs of hemolysis. To explore independent association of variables correlated with outcome (*ie*, mortality and VAP) we performed stepwise logistic multivariable analyses.

Differences in vascular resistance, VAP proportion, and mortality were tested using a nonparametric test for trend across tertiles of indirect signs of hemolysis. Univariate correlations between continuous variables were reported using the Spearman correlation coefficient (*ρ*).

Statistical significance was reached when the *p*-value was <0.05 (two-tailed). Statistical analyses were performed using STATA-14/MP (StataCorp LP, College Station, TX) and GraphPad Prism 8.3.0 (GraphPad Software, San Diego, CA).

### Time-Course of Indirect Signs of Hemolysis and Hemodynamic Variables During Extracorporeal Membrane Oxygenation Stratified by Mortality

We further explored the time-course of physiological variables (*ie*, COHb, haptoglobin, PVR, and SVR) during ECMO after stratification by ICU mortality. Statistical analyses on time-course of physiological variables during ECMO were performed as previously described^23^ and carried out by SAS 9.4 statistical package.

Comprehensive details on statistical analyses are reported in Supplemental Digital Content, http://links.lww.com/ASAIO/B310.

## Results

### Characterization of the Patient Population Before Extracorporeal Membrane Oxygenation Cannulation

From October 2003 to May 2019, we managed a total of 249 patients with VV-ECMO for respiratory support. As per inclusion criteria, 147 ARDS patients undergoing VV-ECMO were included in the final analysis (sFigure 1, Supplemental Digital Content, http://links.lww.com/ASAIO/B310). All but one of the 147 patients had the drainage cannula positioned in a femoral vein. The right internal jugular vein was used in one case. The return cannula was positioned in 130/147 patients in the contralateral femoral vein, in 16 patients in the right internal jugular vein, and in one patient in a femoral vein. Of 147 patients, 47 patients (32%) did not survive to ICU discharge. Baseline, clinical and laboratory characteristics of the study population before ECMO cannulation stratified by ICU survival are described in Table 1. Before ECMO initiation, indirect signs of hemolysis were significantly different between nonsurvivors and survivors with higher levels of COHb (169 [71–197] *vs.* 124 [41–146] mg/dl, *p* = 0.003) and lower levels of haptoglobin (208 [99–238] *vs.* 249 [145–354] mg/dl, *p* = 0.010) in patients who did not survive at ICU discharge (Table 1).

**Table 1.** Baseline, Clinical and Laboratory Characteristics of the Study Population Before Veno-Venous ECMO and Clinical Outcomes Stratified by ICU Survival

Baseline Characteristics	All, n = 147	Survivors, n = 100 (68%)	Nonsurvivors, n = 47 (32%)	*p*
Baseline characteristics				
Study year, median (IQR)	2014 (2011–2017)	2015 (2011–2017)	2013 (2011–2016)	0.089
Gender (M/F), n (%)	92/55 (63/37)	61/39 (61/39)	31/16 (66/34)	0.562
Age (years), median (IQR)	51 (41–59)	51 (41–57)	56 (45–63)	0.004
BMI (kg/m^2^), median (IQR)	26.1 (23.9–30.9)	26.2 (23.9–31.8)	26.1 (22.9–28.3)	0.167
Etiology of respiratory failure, n (%)				
Bacterial	62	41 (41)	21 (45)	0.673
Viral	58	43 (43)	15 (32)	0.200
Trauma	6	5 (5)	1 (2)	0.665
Autoimmune	12	9 (9)	3 (6)	0.752
Aspiration pneumonia	2	1 (1)	1 (2)	0.539
TBC	3	1 (1)	2 (4)	0.240
Other	4	0 (0)	4 (9)	0.010
Clinical characteristics before ECMO cannulation				
Days of mechanical ventilation before ECMO, median (IQR)	3 (1–7)	2.3 (1–5)	6 (2–11)	<0.001
Bacteremia pre-ECMO, n (%)	14 (9.5)	6 (6.0)	8 (17.0)	0.034
Illness severity before ECMO cannulation				
PRESERVE index, mean ± SD	3.5 ± 2.2	2.9 ± 2.0	4.7 ± 1.9	0.230
RESP score, median (IQR)	3 (1–5)	4 (2–5)	1 (–1 to 3)	0.307
SOFA respiratory, median (IQR)	4 (4–4)	4 (4–4)	4 (4–4)	0.134
SOFA cardiovascular, median (IQR)	2 (0–4)	2 (0–4)	3 (0–4)	0.886
SOFA coagulation, median (IQR)	1 (0–2)	0 (0–1)	1 (0–2)	0.010
SOFA renal, median (IQR)	0 (0–1)	0 (0–1)	0 (0–4)	0.509
SOFA liver, median (IQR)	0 (0–1)	0 (0–1)	0 (0–2)	0.137
SOFA neuro, median (IQR)	0 (0–0)	0 (0–0)	0 (0–0)	0.165
VIS score, median (IQR)	4.8 (0–20)	3 (0–20)	5 (0–16.3)	0.698
Respiratory system compliance (ml/cm H_2_O), median (IQR)	27 (19–36)	29 (23–38)	20 (14–31)	0.001
PaO_2_/FiO_2_ (mm Hg), median (IQR)	72 (58–90)	75 (56–94)	68 (59–85)	0.386
CRRT, n	27 (18.4)	13 (13.0)	14 (29.8)	0.014
Laboratory markers before ECMO cannulation				
Lactate (mM/L), median (IQR)	2.0 (1.3–3.4)	2.0 (1.3–2.9)	2.3 (1.6–5.0)	0.017
CRP (mg/dL), median (IQR)	23 (12–33)	23 (13–33)	23 (7–30)	0.307
Iron (µg/dL), median (IQR)	40 (27–65)	36 (27–55)	61 (28–90)	0.024
Ferritin (ng/mL) median (IQR)	1,430 (678–2,751)	1,367 (657–2,759)	1,725 (846–2,651)	0.594
Transferrin (mg/dL), median (IQR)	109 (82–140)	115 (89–147)	99 (71–130)	0.057
D-dimer (µg/L), median (IQR)	3,079 (1,510–6,260)	3,048 (1,568–5,707)	3,280 (1,389–6,951)	0.904
Fibrinogen (mg/dl), median (IQR)	567 (390–772)	579 (403–810)	552 (367–712)	0.111
COHb (%), median (IQR)	1.2 (0.4–1.5)	1.1 (0.3–1.4)	1.5 (0.5–1.9)	<0.001
COHb (mg/dl), median (IQR)	130 (46–171)	124 (41–146)	169 (71–197)	0.003
Hb (g/dl), median (IQR)	11 (10.2–11.9)	11.1 (10.3–12.1)	10.5 (10.0–11.6)	0.077
Haptoglobin (mg/dL), median (IQR)	221 (134–323)	249 (145–354)	208 (99–238)	0.010
LDH (IU/L), median (IQR)	529 (398–791)	547 (414–803)	483 (370–660)	0.287
Creatinine (mg/dl), median (IQR)	1 (0.7–1.6)	1 (0.7–1.6)	0.9 (0.6–2.0)	0.401

Continuous data were expressed as mean ± SD and median (IQR) as appropriate. Categorical data were expressed as frequency (%).

BMI, body mass index; ECMO, extra corporeal membrane oxygenation; F, female; IQR, interquartile range; LDH, lactate dehydrogenase; M, male; PRESERVE, PRedicting dEath for SEvere ARDS on VV-ECMO; RESP, respiratory extracorporeal membrane oxygenation survival prediction; SD, standard deviation; SOFA, Sequential Organ Failure Assessment; TBC, mycobacterium tuberculosis pulmonary infection; VIS, vaso-inotropic score.

### Clinical and Laboratory Characteristics During Extracorporeal Membrane Oxygenation

**Figure 1.**
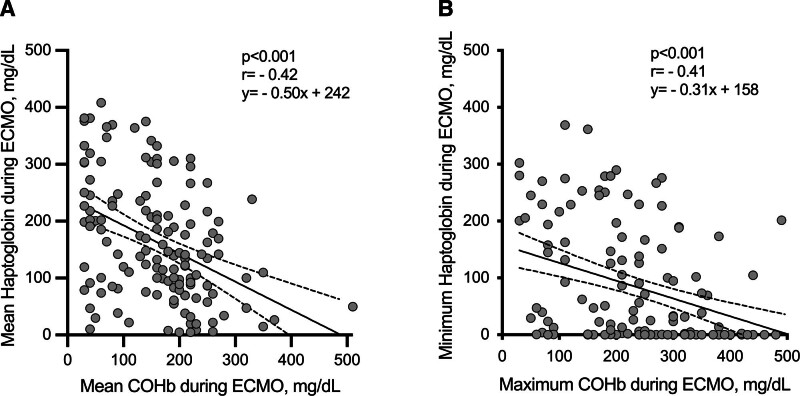
Linear regression between mean COHb and mean haptoglobin (**A**) and between maximum COHb and minimum haptoglobin levels (**B**) during ECMO. COHb, carboxyhemoglobin; ECMO, extracorporeal membrane oxygenation.

On the first day of ECMO, the shunt fraction of the natural lung was significantly higher in nonsurvivors (65% ± 17% *vs.* 57% ± 15%, *p* = 0.003). Patients who died in ICU were on ECMO for longer time (median 27 [11–52] *vs.* 14 [9–22] days, *p* = 0.001) and were transfused with greater volume of red blood cells (301 [212–392] *vs.* 200 [143–271] ml/day, *p* < 0.001) compared to survivors. During ECMO, average concentration of COHb (210 [110–250] *vs.* 155 [60–200] mg/dl, *p* < 0.001) and haptoglobin (106 [65–142] *vs.* 186 [89–267] mg/dl, *p* < 0.001) were significantly different between nonsurvivors and survivors (Table 2). Average COHb and haptoglobin during ECMO were negatively correlated (*r* = −0.42, *p* < 0.001) (Figure 1A) as were maximum COHb and minimum Haptoglobin during ECMO (*r* = −0.41, *p* < 0.001) (Figure 1B).

**Table 2.** Clinical and Laboratory Characteristics of the Study Population During Veno-Venous ECMO and Outcomes Stratified by ICU Survival

	All, n = 147	Survivors, n = 100 (68%)	Nonsurvivors, n = 47 (32%)	*p*
ECMO and laboratory characteristics, average data during ECMO				
Drainage cannula size, French 21 23 24 25 27	6 (4.1) 61 (41.8) 1 (0.7) 77 (52.7) 1 (0.7)	3 (3) 51 (51) 1 (1) 44 (44) 1 (1)	3 (6.5) 10 (21.7) 0 (0) 33 (71.8) 0 (0)	0.012
Return cannula size, French 17 19 21 23 25	2 (1.4) 12 (8.2) 68 (46.6) 61 (41.8) 3 (2.0)	1 (1) 9 (9) 47 (47) 42 (42) 1 (1)	1 (2.2) 3 (6.5) 21 (45.7) 19 (41.3) 2 (4.3)	0.683
First shunt, day 1 of ECMO (%), mean ± SD	59 ± 16	57 ± 15	65 ± 17	0.003
Mean ECMO blood flow (L/min), median (IQR)	3.5 (3.1–3.8)	3.4 (3.1–3.8)	3.6 (3.2–3.9)	0.122
Mean ECMO drainage pressure (mm Hg), median (IQR)	−39 (−47 to −31)	−39 (−47 to −31)	−39 (−48 to −28)	0.915
Mean daily RBC transfusion, volume (ml), median (IQR)	219 (149–312)	200 (143–271)	301 (212–392)	<0.001
Days of ECMO, n, median (IQR)	16 (10–27)	14 (9–22)	27 (11–52)	0.001
Circuits, n, median (IQR)	2 (1–4)	2 (1–4)	2 (1–6)	0.457
Average circuit duration, days, median (IQR)	6 (5–9)	6 (5–8)	9 (5–13)	<0.001
Mean hemoglobin (g/dl), median (IQR)	10.6 (10.2–10.9)	10.5 (10.3–10.9)	10.6 (10.2–10.9)	0.808
D-dimer (ng/ml), median (IQR)	4,347 (3,067–7,051)	4,775 (3,389–7,599)	3,869 (2,222–5,842)	0.023
Mean creatinine (mg/dl), median (IQR)	0.8 (0.5–1.2)	0.9 (0.6–1.3)	0.8 (0.5–1.2)	0.144
Mean PEEP (cm H_2_O), median (IQR)	15.3 (13.0–16.6)	15.8 (13.7–17.0)	14.4 (11.5–15.9)	0.005
Mean airway pressure (cm H_2_O) median (IQR)	18.7 (16.7–20.4)	18.9 (17.0–20.4)	18.4 (16.1–20.4)	0.624
Mean peak inspiratory pressure (cm H_2_O), median (IQR)	28.6 (25.5–31.0)	28.3 (25.3–30.6)	28.9 (26.6–33.1)	0.047
Indirect signs of hemolysis and hemodynamics, average data during ECMO				
Mean COHb (%), median (IQR)	1.6 (0.8–2.1)	1.4 (0.6–1.8)	2.0 (1.2–2.3)	<0.001
Mean COHb (mg/dl), median (IQR)	160 (80–220)	155 (60–200)	210 (110–250)	<0.001
Max COHb (%), median (IQR)	2.2 (1.5–3.0)	2.0 (1.2–2.5)	2.9 (2.3–3.7)	<0.001
Max COHb (mg/dl), median (IQR)	240 (150–320)	210 (110–270)	300 (230–390)	<0.001
Mean LDH (IU/L), median (IQR)	433 (358–563)	426 (364–563)	455 (331–736)	<0.001
Mean haptoglobin (mg/dl), median (IQR)	142 (83–228)	186 (89–267)	106 (65–142)	<0.001
Min haptoglobin (mg/dl), median (IQR)	24 (0.1–152)	47 (0.6–201)	0.2 (0.1–39)	<0.001
Mean HR (beats/min), mean ± SD	95 ± 13	94 ± 13	98 ± 14	0.090
Mean CO (L/min), mean ± SD	7.9 ± 1.5	8.0 ± 1.4	7.7 ± 1.8	0.222
Mean mPAP (mm Hg), mean ± SD	28 ± 5	27 ± 5	29 ± 6	0.012
Mean PAOP (mm Hg), mean ± SD	13 ± 2	13 ± 2	12 ± 3	0.230
Mean PVR (dyn/sec/cm^−5^), median (IQR)	149 (123–180)	144 (121–169)	170 (132–225)	0.004
Mean MAP (mm Hg), mean ± SD	79 ± 7	81 ± 6	75 ± 6	<0.001
Mean CVP (mm Hg), mean ± SD	11 ± 2	11 ± 2	11 ± 3	0.453
Mean SVR (dyn/sec/cm^−5^), median (IQR)	706 (633–843)	712 (651–825)	660 (581–880)	0.214
Outcomes				
ICU length of stay (days), median (IQR)	33 (22–54)	33 (22–48)	37 (18–77)	0.341
Ventilator free days (days), median (IQR)	0 (0–5)	0 (0–10)	0 (0–0)	<0.001
Creatinine max (mg/dl), median (IQR)	1.3 (0.8–2.0)	1.3 (0.9–2.3)	1.3 (0.8–1.8)	0.678
CRRT, n (%)	63 (42.9)	35 (35.0)	28 (59.6)	0.005
CRRT during ECMO, n (%)	38 (25.9)	23 (23.0)	15 (31.9)	0.250
Bacteremia during ECMO, n (%)	38 (25.9)	18 (18.0)	20 (43)	0.002
VAP during ECMO n (%)	50 (34.0)	25 (25.0)	25 (53.2)	0.001
Aspergillosis, n (%)	19 (12.9)	5 (5.0)	14 (30)	<0.001

Continuous data were expressed as mean ± SD and median (IQR) as appropriate. Categorical data were expressed as frequency (%).

COHb, carboxyhemoglobin; CO, cardiac output; CRRT, continuous renal replacement therapy; CVP, central venous pressure; ECMO, extracorporeal membrane oxygenation; ICU, intensive care unit; LDH, lactate dehydrogenase; MAP, mean arterial pressure; mPAP, mean pulmonary artery pressure; PAOP, pulmonary artery occlusion pressure; PEEP, positive end-expiratory pressure; PVR, pulmonary vascular resistance; RBC, red blood cell; SVR, systemic vascular resistance; VAP, ventilator-associated pneumonia.

In univariate analyses, we observed that RBC transfusion, days on ECMO, number of ECMO circuits, average circuit duration, and average d-Dimer levels during ECMO were all positively associated with higher average levels of COHb during ECMO. Red blood cell transfusion, days on ECMO, number of ECMO circuits, average circuit duration were all negatively correlated with a lower average level of haptoglobin during the ECMO (sTable1, Supplemental Digital Content, http://links.lww.com/ASAIO/B310).

### Association Between Indirect Signs of Hemolysis and Hemodynamics During Extracorporeal Membrane Oxygenation

Nonsurvivors had higher PVR during ECMO compared to survivors (170 [132–225] *vs.* 144 [121–169] dyn/sec/cm^−5^, *p* = 0.004). In contrast, SVR did not differ between the groups (Table 2).

**Figure 2.**
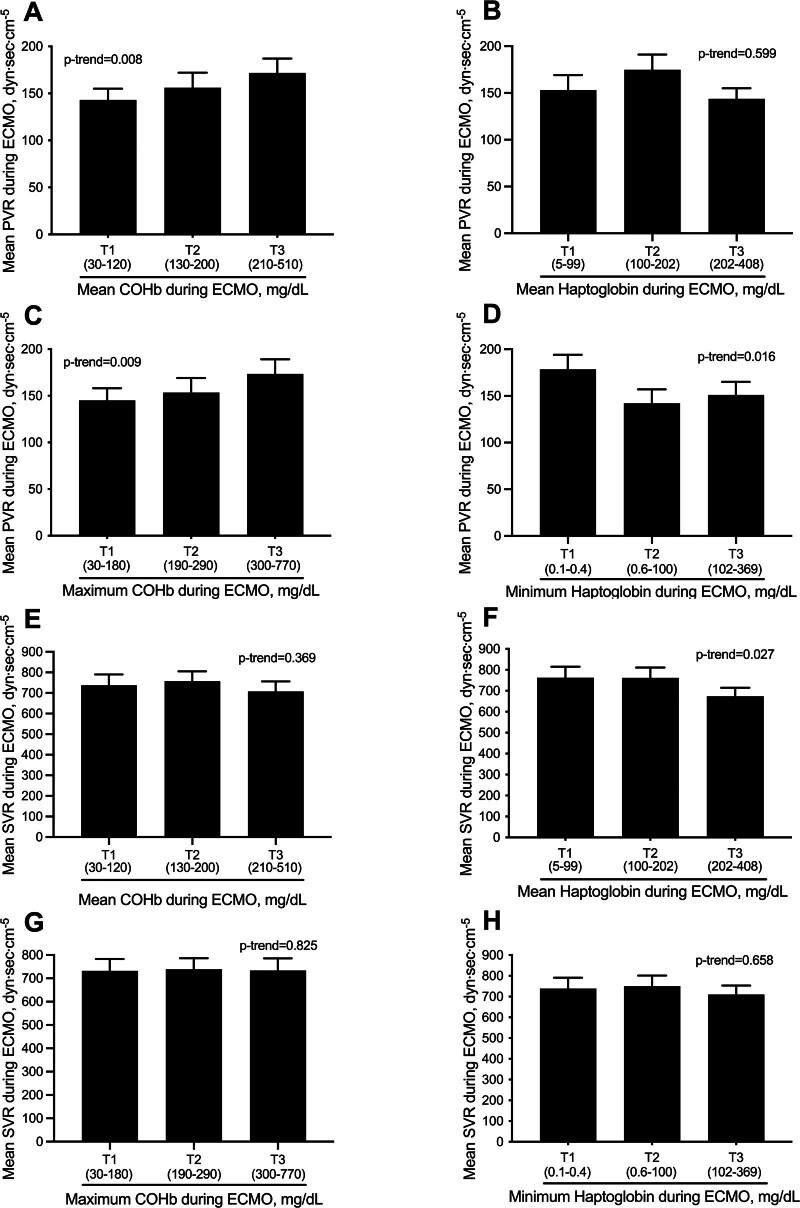
Pulmonary vascular resistance stratified by tertiles (T1, T2, and T3) of mean COHb and mean haptoglobin (**A, B**) and by tertiles of maximum COHb and minimum haptoglobin (**C, D**) during ECMO. Systemic vascular resistance stratified by tertiles (T1, T2, and T3) of mean COHb and mean Haptoglobin (**E, F**) and by tertiles of maximum COHb and minimum haptoglobin (**G, H**) during ECMO. COHb, carboxyhemoglobin; ECMO, extracorporeal membrane oxygenation; PVR, pulmonary vascular resistance; SVR, systemic vascular resistance.

Levels of PVR and SVR stratified by tertiles of indirect signs of hemolysis are shown in Figure 2. Higher average COHb levels (*p*-trend = 0.008) during ECMO—but not lower average haptoglobin levels—were associated with increased PVR levels (Figure 2, A and B). Higher maximum COHb levels (*p*-trend = 0.009) during ECMO and lower minimum haptoglobin levels were associated with increased PVR levels (Figure 2D). Higher mean COHb levels during ECMO did not significantly correlate with lower SVR levels (Figure 2E), whereas lower average haptoglobin levels did (*p* = 0.027) (Figure 2F). Maximum COHb levels and minimum haptoglobin levels did not correlate with SVR. Linear correlations between average levels of COHb and haptoglobin, maximum levels of COHb, and minimum levels of haptoglobin with PVR and SVR during ECMO were reported in sFigure2, Supplemental Digital Content, http://links.lww.com/ASAIO/B310.

### Study Outcomes

Patients who died in ICU had a higher need of CRRT and presented more relevant infective complications compared to survivors. Specifically, bacteremia, pulmonary infection by aspergillosis and VAP occurred more often in nonsurvivors (Table 2). We now explore the variables independently associated with the study outcomes: 1) VAP; and 2) ICU mortality.

#### Predictors of VAP

**Figure 3.**
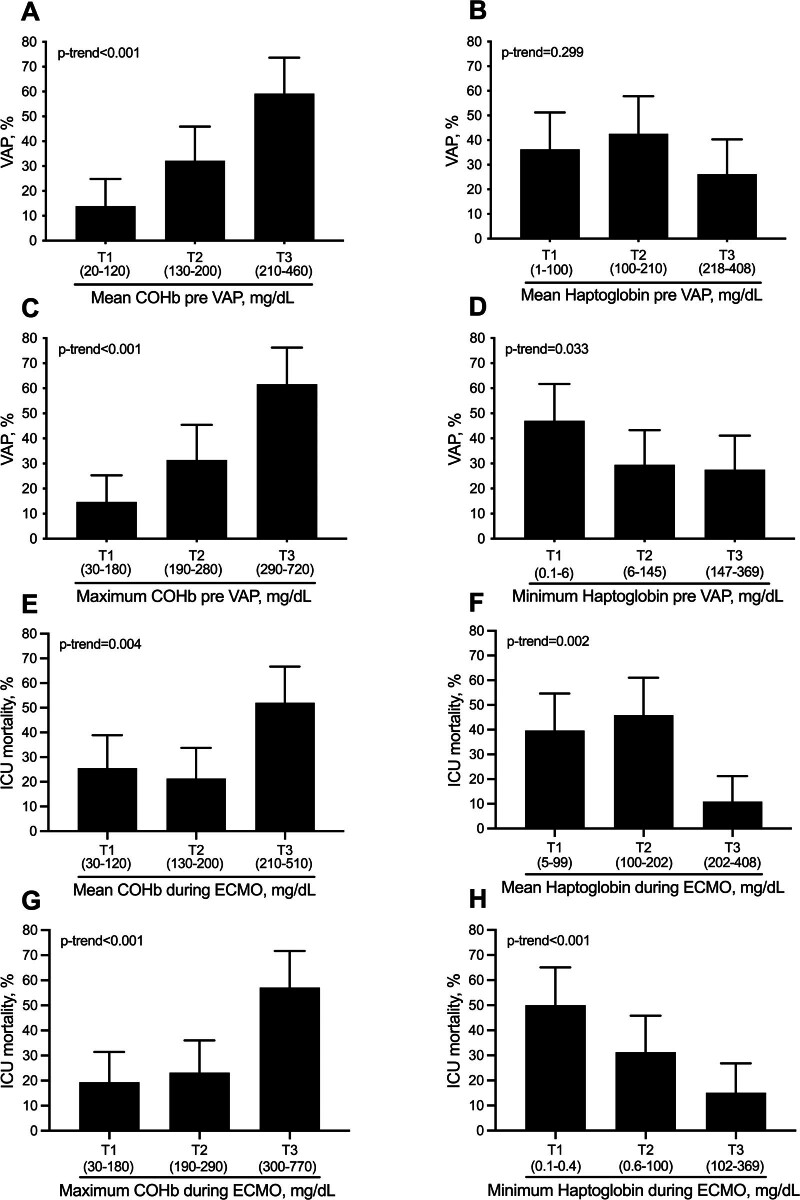
Ventilator-associated pneumonia stratified by tertiles (T1, T2, and T3) of mean COHb and mean Haptoglobin (**A, B**) and by tertiles of maximum COHb and minimum haptoglobin (**C, D**) before VAP. ICU mortality stratified by tertiles (T1, T2, and T3) of mean COHb and mean Haptoglobin (**E, F**) and by tertiles of maximum COHb and minimum haptoglobin (**G, H**) during ECMO. COHb, carboxyhemoglobin; ECMO, extracorporeal membrane oxygenation; ICU, intensive care unit; VAP, ventilator-associated pneumonia.

Increasing average COHb levels during ECMO before VAP by tertiles were positively correlated with VAP onset (*p*-trend, *p* < 0.001) (Figure 3A). Decreasing average haptoglobin levels during ECMO before VAP by tertiles did not correlate with VAP onset (Figure 3B). Increasing maximum levels of COHb (Figure 3C) and decreasing minimum levels of haptoglobin (Figure 3D) during ECMO before VAP both correlated with VAP onset.

To evaluate whether indirect signs of hemolysis were independently associated with the risk of VAP, we first explored all potential confounders of VAP in a univariate analysis (sTable2, Supplemental Digital Content, http://links.lww.com/ASAIO/B310). Carboxyhemoglobin levels before ECMO cannulation and as average levels before VAP showed unadjusted association with VAP onset. Other associated variables included year of ECMO, BMI, transferrin levels before ECMO, days of mechanical ventilation, and respiratory system compliance before ECMO.

As COHb and haptoglobin were significantly correlated (Figure 1), we tested the role of the two markers of hemolysis in separate multivariable models.

Average and maximum levels of COHb during ECMO before VAP significantly predicted VAP onset after adjusting for clinically meaningful confounders (BMI, the year of admission, transferrin before ECMO, COHb before ECMO, and respiratory system compliance) (Table 3A). No independent association between haptoglobin and VAP was observed after adjusting for confounders (Table 3B). Of note, the transferrin levels measured before ECMO cannulation showed an independent negative association with VAP onset during ECMO.

**Table 3.** Adjusted Predictors of VAP in a Stepwise Multivariable Model

	VAP Onset			VAP Onset		
Variable	OR	95% CI	*p*-Value	OR	95% CI	*p*-Value
A) Adjusted predictors of VAP in a stepwise multivariable model and exploring the role of COHb						
Respiratory system compliance before ECMO cannulation, mL/cmH2O	0.94	0.90–0.98	0.004	0.94	0.90–0.98	0.003
Transferrin before ECMO cannulation, mg/dL	0.99	0.97–1.00	0.016	0.98	0.97–1.00	0.011
Mean COHb during ECMO before VAP, mg/dL	1.01	1.00–1.01	0.004	—	—	—
Maximum COHb during ECMO before VAP, mg/dL				1.01	1.00–1.01	0.006
B) Adjusted predictors of VAP in a stepwise multivariable logistic regression model and exploring the role of haptoglobin						
Respiratory system compliance before ECMO cannulation, mL/cmH2O	0.93	0.89–0.97	0.001	0.93	0.89–0.97	0.001
Transferrin before ECMO cannulation, mg/dL	0.99	0.98–1.00	0.020	0.99	0.97–1.00	0.010
Mean haptoglobin during ECMO before VAP, mg/dL	1.00	0.99–1.00	0.644	—	—	—
Minimum haptoglobin during ECMO before VAP, mg/dL				1.00	0.99–1.00	0.152

CI, confidence interval; COHb, carboxyhemoglobin; ECMO, extracorporeal membrane oxygenation; OR, odds ratio; VAP, ventilator associated pneumonia.

#### Predictors of mortality

Increasing average levels of COHb (Figure 3E) and decreasing average levels of haptoglobin (Figure 3F) by tertiles during ECMO were positively correlated with ICU mortality. Similarly, this was confirmed by exploring increasing levels of maximum COHb (Figure 3G) and decreasing levels of minimum haptoglobin (Figure 3H).

To test whether indirect signs of hemolysis were independently associated with mortality, we first explored all potential confounders of mortality in a univariate analysis (sTable3, Supplemental Digital Content, http://links.lww.com/ASAIO/B310). Carboxyhemoglobin and haptoglobin levels before ECMO cannulation and as average levels during ECMO showed unadjusted association with mortality

Other associated variables included age, days of mechanical ventilation before ECMO, bacteremia and respiratory system compliance before ECMO, and other laboratory and clinical variables (*eg*, hemodynamics and ventilation pressure during ECMO).

As COHb and haptoglobin were significantly correlated to each other (Figure 1), we studied the role of the two markers of hemolysis in separate multivariable models.

In both multivariate models either mean or maximum COHb levels (Table 4A) and either mean or minimum haptoglobin levels (Table 4B) were confirmed as independent predictors of mortality after adjusting for clinically meaningful confounders (PRESERVE, respiratory system compliance and iron at ECMO cannulation, average PEEP, PVR, and SVR during ECMO, COHb, and haptoglobin before ECMO). Of note, the iron pathway explored before ECMO cannulation unveiled the independent positive association of iron levels and ICU mortality rate.

**Table 4.** Adjusted Predictors of Mortality in a Stepwise Multivariable Model

	Mortality			Mortality		
Variable	OR	95% CI	*p*-Value	OR	95% CI	*p*-Value
A) Adjusted predictors of mortality in a stepwise multivariable logistic regression analysis and exploring the role of COHb						
PRESERVE index, unit increase	1.78	1.35–2.34	<0.001	1.70	1.29–2.25	<0.001
Iron before ECMO cannulation, µg/dL	1.01	1.00–1.03	0.037	1.01	1.00–1.03	0.036
Mean PVR during ECMO, dyn/sec/cm^−5^	1.02	1.01–1.03	0.003	1.02	1.00–1.03	0.007
Mean SVR during ECMO, dyn/sec/cm^−5^	1.00	0.99–1.00	0.016	1.00	0.99–1.00	0.024
Mean COHb during ECMO, mg/dL	1.01	1.00–1.01	0.008	—	—	—
Maximum COHb during ECMO, mg/dL				1.01	1.00–1.01	0.002
B) Adjusted predictors of mortality in a stepwise multivariable logistic regression analysis and exploring the role of haptoglobin						
PRESERVE index, unit increase	1.73	1.28–2.34	<0.001	1.61	1.23–2.11	<0.001
Iron before ECMO cannulation, µg/dL	1.01	1.00–1.02	0.088	1.01	1.00–1.02	0.062
Mean PVR during ECMO, dyn/sec/cm^−5^	1.03	1.01–1.03	<0.001	1.02	1.01–1.04	<0.001
Mean SVR during ECMO, dyn/sec/cm^−5^	0.99	0.99–1.00	0.001	0.99	0.99–1.00	0.006
Mean haptoglobin during ECMO, mg/dL	0.99	0.99–1.00	0.001	—	—	—
Minimum haptoglobin during ECMO, mg/dL				0.99	0.99–1.00	0.014

CI, confidence interval; COHb, carboxyhemoglobin; OR, odds ratio; PRESERVE, PRedicting dEath for SEvere ARDS on VV-ECMO; PVR, pulmonary vascular resistance; VV-ECMO, . veno-venous extracorporeal membrane oxygenation.

### Longitudinal Analyses: Time-Course of Carboxyhemoglobin, Haptoglobin, and Vascular Resistances During Extracorporeal Membrane Oxygenation Stratified by Mortality

**Figure 4.**
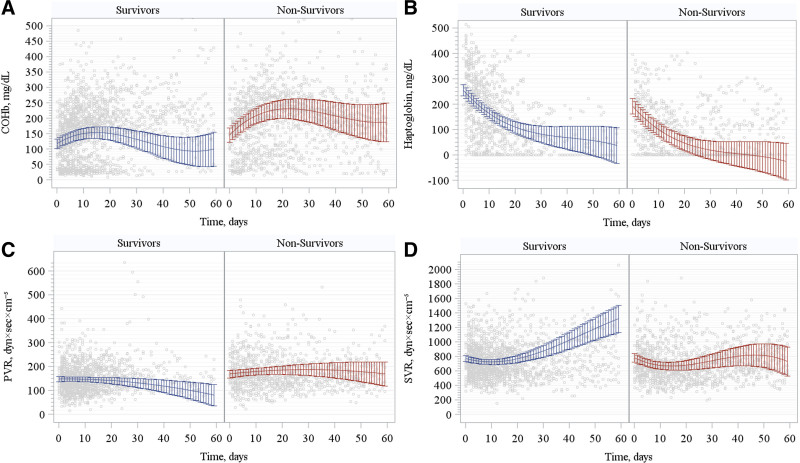
Trends of indirect signs of hemolysis (COHb (**A**) and haptoglobin (**B**)) and vascular resistances (PVR (**C**) and SVR (**D**)) during ECMO over time. The polynomial maximum likelihood multilevel model results are represented by the blue line (survivors) and the red line (nonsurvivors) with 95% confidence interval (whiskers). Gray dots represent experimental data. *p* < 0.001 *vs.* nonsurvivors in all four panels. COHb, carboxyhemoglobin; PVR, pulmonary vascular resistance; SVR, systemic vascular resistance.

We further explored differences in the trajectories of indirect signs of hemolysis during ECMO over 60 day follow-up stratified by survivors and nonsurvivors. Although COHb showed a trend towards higher levels over time (Figure 4A), haptoglobin showed a trend towards lower levels over time (Figure 4B) in nonsurvivors as compared to survivors.

About hemodynamics, we observed a trend to higher PVR levels during ECMO (Figure 4C), whereas a trend to a lower SVR levels during ECMO (Figure 4D) in nonsurvivors as compared with survivors. A comprehensive description of longitudinal models at 60 day follow-up was reported in Supplemental Digital Content, http://links.lww.com/ASAIO/B310.

## Discussion

In this observational retrospective cohort study that included a selected population of critically ill patients with severe ARDS undergoing VV-ECMO, we observed that signs of hemolysis are independently associated with patient outcomes and are associated with the modulation of both systemic and PVR.

The main findings of our clinical investigation can be summarized as follows:

- higher and lower levels of COHb and haptoglobin were associated with higher RBC transfusion requirements, longer ECMO, and circuit duration;
- higher levels of COHb were associated with pulmonary vasoconstriction, whereas lower levels of haptoglobin were associated with both pulmonary and systemic vasoconstriction;
- increasing levels of COHb were associated with a higher incidence of VAP, robust to multivariable adjustments;
- decreasing levels of haptoglobin were associated with a higher incidence of VAP (not confirmed after covariate adjustments in multivariable models);
- increasing levels of COHb and decreasing levels of haptoglobin were both independently correlated with mortality; and
- in longitudinal models, COHb and PVR showed higher trajectories in nonsurvivors, whereas haptoglobin and SVR showed higher trajectories in nonsurvivors.

This study includes a patient population with severe ARDS undergoing ECMO in a tertiary referral hospital. It provides comprehensive information about daily hemodynamics of these patients including the evaluation of PVR because all patients were monitored with a pulmonary artery catheter along with detailed information about daily levels of haptoglobin and COHb during ECMO.

### Variables Associated With Indirect Signs of Hemolysis During Extracorporeal Membrane Oxygenation Support

In this study, we explored the association between average levels of COHb and haptoglobin during the ICU stay and physiologic and outcome variables in severe ARDS patients undergoing VV-ECMO. We observed that higher amount of RBC transfusion, time spent on ECMO, number of ECMO circuits, higher Dimer levels during ECMO and drainage pressures on ECMO were associated with higher average levels of COHb and lower average haptoglobin levels in plasma during ECMO. These findings confirm previous literature data on the role of the shear forces within the ECMO circuit together with the amount of transfused blood as major contributors to intravascular hemolysis during extracorporeal circulation.^24^

### Correlation Between Indirect Signs of Hemolysis and Pulmonary and Systemic Hemodynamics

Cell-free hemoglobin has been widely investigated for its role in modulating vascular resistance. More than 15 years ago, an elegant mechanistic study by Minneci *et al*.^25^ reported the role of free oxyhemoglobin in increasing both systemic and PVR. Years later, similar findings were described in cardiac surgery patients.^26^ Interestingly, hemolysis is also involved in pulmonary hypertension, a frequent complication of severe ARDS.^27^ To our knowledge, the current study is the first to describe an association of indirect signs of hemolysis such as COHb and haptoglobin with vascular tone in ECMO patients. This finding—although in a retrospective study design—may be a relevant indicator to remind the clinician about CFH induced endothelial dysfunction and its potential effect on perfusion.^28^

### Indirect Signs of Hemolysis, Lung Injury, and Ventilator-Associated Pneumonia During Veno-Venous Extracorporeal Membrane Oxygenation

It has recently been proposed that CFH may play a role in the pathogenesis of lung epithelial and endothelial injury. The potential key players include promotion of inflammation within the airspace, an increase in alveolar permeability caused by alveolar-capillary barrier dysfunction^29^ and oxidative reactions.^30^ Hemolysis related to transfusion of RBCs with prolonged storage duration was associated with increased risk of acute lung injury in septic patients^31^ and risk of secondary infections.^32^

Interestingly, Earley *et al.*^33^ reported that a decrease in the volume of blood transfusion in trauma patients was associated with a reduced onset of ventilator-associated pneumonia suggesting that lower levels of hemolysis due to transfusion reduce triggering of lung injury.

In our study, we observed a linear increase in the risk of VAP with increasing levels of COHb and with depleted levels of haptoglobin, the primary endogenous scavenger of CFH. However, only COHb had an independent association with VAP incidence. Moreover, some investigators reported haptoglobin among potential genes differently expressed in patients with or without VAP.^34^ Interestingly, lower levels of transferrin before ECMO cannulation were independently associated with VAP onset, suggesting a potential role of the iron pathway involved in inflammatory and oxidative reactions in the onset of VAP.^30,35^ Our findings suggest that decreasing the hemolysis levels and modulating the iron pathway may contribute on preventing VAP onset in ECMO.

### Indirect Signs of Hemolysis, Lung Injury, and Intensive Care Unit Mortality During Veno-Venous Extracorporeal Membrane Oxygenation

We further investigated the association of COHb and haptoglobin with ICU mortality. Similar to the association with VAP, higher COHb was associated with ICU mortality—as represented by increasing frequency of death for increasing COHb levels by tertile. Again, low haptoglobin levels showed a higher proportion of mortality in our population. We further explored trajectories of indirect biomarkers of hemolysis over time during ECMO between survivors and nonsurvivors and observed higher daily levels of COHb and lower daily levels of haptoglobin in patients who did not survive.

Furthermore, the iron pathway showed an independent association as well with ICU mortality in VV-ECMO patients suggesting a potential role of the inflammatory and oxidative stress related to free iron. These findings are in line with data reported by Tacke *et al.*^35^ on the role of iron metabolism on outcome in critically ill patients and suggest the potential of iron chelating therapy in the critical illness.

Our data confirm the associative findings of Omar *et al.*^36^ who reported that higher hemolysis—defined with CFH >50 mg/dl—at 24 h after ECMO cannulation was predictive of mortality in patients undergoing ECMO. Recently, Cholette *et al.*^18^ reported that hemolysis was independently associated with mortality in a pediatric population undergoing cardiac surgery; both high levels of CFH and low levels of haptoglobin, were significantly associated with outcomes. These findings suggest the need for prospective studies to clarify first the role of COHb and haptoglobin as mediators versus markers of outcomes and second whether, potentially, patients undergoing subclinical and chronic hemolysis—such as patients undergoing ECMO—might benefit by administration of CFH scavengers such as haptoglobin^37^ or nitric oxide.^38^

This study has some limitations that should be acknowledged. First, we only measured hemolysis indirectly because direct measures of hemolysis such as plasma CFH were not available. In addition, this study is retrospective, with the study question defined after the data collection. However, all the variables were systematically collected in a standard way in patients undergoing VV-ECMO, immediately verified and stored in a database. The study includes patients evaluated in our ICU over a long period of time so unknown confounders might have been in play. Further, as the number of VV-ECMO patients managed at our center increased over the years, we cannot exclude that this may have had an impact on our practice and on the management of hemolysis during ECMO.

## Conclusions

In patients undergoing VV-ECMO for severe ARDS, indirect signs of hemolysis such as plasma levels of COHb and haptoglobin show an association with pulmonary and SVR; COHb levels during ECMO before VAP independently predict VAP; both COHb and haptoglobin levels during ECMO independently predict mortality; the iron pathway is associated with VAP onset (*ie*, transferrin levels before ECMO) and mortality (*ie*, iron levels before ECMO) after adjustment for major confounders in multivariable models.

Further studies may prospectively validate our findings by the measurement of CFH and determine whether treatments aimed at replenishing haptoglobin levels or at lowering iron levels might be beneficial in ARDS patients undergoing ECMO who are often exposed to chronic and subclinical hemolysis.

## Acknowledgments

The authors are indebted to all the staff physicians, the nursing staff, and all the healthcare workers who commit daily dedication, time, and care to all of our patients of the Intensive Care Unit “Rianimazione Generale,” Fondazione IRCCS San Gerardo, Monza, Italy.

## Supplementary Material
