# Supplementary material for: Signs of Hemolysis Predict Mortality and Ventilator Associated Pneumonia in Severe Acute Respiratory Distress Syndrome Patients Undergoing Veno-Venous Extracorporeal Membrane Oxygenation
Source: ASAIO J. 2024 Jul 30;71(1):82–91. doi: 10.1097/MAT.0000000000002278 (PMC11670904; doi:10.1097/MAT.0000000000002278)
Supplement: Supplementary file 1 [file mat-71-082-s001.pdf]

Supplemental material

Signs of hemolysis predict mortality and ventilator associated pneumonia in severe Acute Respiratory Distress Syndrome patients undergoing veno-venous Extracorporeal Membrane Oxygenation.

Emanuele Rezoagli[1,2],* MD, Michela Bombino[2],* MD, PhD, Lorraine B. Ware[3,4], MD, Eleonora Carlesso[5], PhD, Roberto Rona[2], MD, Giacomo Grasselli[5,6], MD, Antonio Pesenti[5,6], MD, Giacomo Bellani[7,8], MD, PhD,[#] Giuseppe Foti[1,2], MD[#]

Affiliations:

[1]School of Medicine and Surgery, University of Milan-Bicocca, Via Cadore 48, 20900, Monza (MB), Italy

[2]Department of Emergency and Intensive Care, Fondazione IRCCS San Gerardo dei Tintori, Via Giambattista Pergolesi 33, 20900, Monza (MB), Italy

[3]Allergy, Pulmonary, and Critical Care Medicine, Department of Medicine, Vanderbilt University Medical Center, Nashville, TN, United States

[4]Department of Pathology, Microbiology and Immunology, Vanderbilt University Medical Center, Nashville, TN, United States

[5]Department of Medical Physiopathology and Transplants, University of Milan, Via Della Commenda 16, 20122, Milano (MI), Italy

[6]Department of Anesthesia, Critical Care and Emergency, Fondazione IRCCS Ca' Granda - Ospedale Maggiore Policlinico, Via Francesco Sforza 35, 20122, Milan (MI), Italy

[7]Centre for Medical Sciences - CISMed, University of Trento, Santa Chiara Regional Hospital, Trento, Italy

[8]Anesthesia and Intensive Care, Santa Chiara Regional Hospital, Trento, Italy

*co-first authors

#co-senior authors

Corresponding Author:

Emanuele Rezoagli, MD, PhD

School of Medicine and Surgery

University of Milan-Bicocca

Via Cadore 48, Monza (MB), Italy

emanuele.rezoagli@unimib.it

Methods

Statistical Analysis

Normality of data distribution was tested using the Shapiro-Wilk test. Continuous variables were expressed as mean ± standard deviation (SD) or as median [interquartile range] according to the data distribution. Categorical data were described as proportion (percentage). The patient population was stratified into ICU survivors and non-survivors. Differences between continuous data between survivors and non-survivors were tested using unpaired Student's T-test or Wilcoxon rank-sum test as appropriate. A difference in categorical data was tested using Chi square or Fisher's exact test. To investigate the role of variables potentially associated with indirect signs of hemolysis we performed univariate linear regression analysis and we reported the coefficient Beta with the 95% CI.

Differences in vascular resistance (PVR and SVR) were tested using non-parametric test for trend across tertiles of indirect signs of hemolysis by using the Cochran–Armitage test.

VAP proportion and mortality were tested using non-parametric test for trend across tertiles of indirect signs of hemolysis by using the Jonckheere–Terpstra test. Indirect signs of hemolysis were explored as average levels of both both peak in COHb (maximum COHb) and average levels of COHb during ECMO; and lowest drop in Haptoglobin (minimum Haptoglobin) and average levels of haptoglobin during ECMO.

In order to explore the presence of variables correlated with outcome (i.e. VAP and mortality) we performed univariate logistic regression analyses and we reported the odds ratio with the 95% CI. To evaluate the presence of independent predictors of outcome, we selected clinically meaningful variables that were significantly associated with outcome at the univariate analysis with a threshold p-value of 0.05 to test in a multivariate logistic regression model. A stepwise

approach was finally used to retain independent variables associated with the outcome using a threshold p-value of 0.05. Indirect signs of hemolysis were tested into these multivariable models by considering either peak in COHb (maximum COHb) or average levels of COHb during ECMO before VAP; and either the lowest drop in Haptoglobin (minimum Haptoglobin) or average levels of haptoglobin during ECMO before VAP when we explored VAP as outcome.

Clinically meaningful significant covariates at the univariate analysis and explored in the multivariable model assessing VAP as outcome were the following ones:

BMI, year of inclusion, transferrin levels before ECMO, compliance of the respiratory system + COHb before ECMO and COHb during ECMO before VAP onset or haptoglobin before ECMO and haptoglobin during ECMO before VAP onset.

Indirect signs of hemolysis were tested into these multivariable models by considering either peak in COHb (maximum COHb) or average levels of COHb during ECMO; and either the lowest drop in Haptoglobin (minimum Haptoglobin) or average levels of haptoglobin during ECMO when we explored mortality as outcome.

Clinically meaningful significant covariates at the univariate analysis and explored in the multivariable model assessing mortality as outcome were the following ones:

PRESERVE, respiratory system compliance, PEEP, PVR, SVR, iron before ECMO + COHb before ECMO and COHb during ECMO or haptoglobin before ECMO and haptoglobin during ECMO.

Univariate correlations between continuous variables within a scatterplot were tested using a linear regression analysis. The degree of association was reported using the Spearman-correlation coefficient (ρ), as appropriate ranging between -1; +1. 95% CI was estimated by bootstrap (1000 replications).

Data missing was limited and no data imputation was performed. Specifically, data availability was present on indirect signs of hemolysis during ECMO in 147/147 patients on COHb and in 137/147 patients about haptoglobin; on study outcomes in 146/147 patients about SVR; in 144/147 patients about PVR; in 147/147 patients about VAP and ICU mortality.

Statistical significance was reached when the p-value was <0.05 (two-tailed). Statistical analyses were performed using STATA-14/MP (StataCorp LP, College Station, TX, USA), GraphPad Prism 8.3.0 (GraphPad Software, San Diego, CA, USA) and Microsoft Excel for Mac 2017, Version 15.32.

Time-course of physiological variables during ECMO

We further explored the time-course of physiological variables (i.e. COHb, haptoglobin, PVR and SVR) during ECMO after stratification by ICU mortality.

Variation of dependent variables over time was modeled according to a polynomial multilevel model (general linear mixed models) with random intercept at subject level and random slope at time (days) level. These models are flexible ways for modeling individual differences, for the examination of time-varying predictor effects and to examine predictors pertaining to groups. The overall pattern of individual differences in time was estimated by linear mixed models using restricted maximum likelihood (REML). The significance of new random effects added to the model (intercept and slope) was evaluated using likelihood ratio tests (LRT). The random intercept model was compared to a null model including only the dependent variable (linear regression) and the random intercept was included if the LRT P value was ≤ 0.100. The random intercept model was used as a null model and compared to the random intercept plus the random

slope null model. The random slope was included if the LRT P value was ≤ 0.100. The model was implemented with a completely general (unstructured) covariance matrix.

The functional form of association between the dependent variables and time was assessed by polynomial models. The best fit was decided by LRT to assess subsequent polynomial models with increasing exponential power (up to 3rd order) using time as a continuous independent variable. To compare these models with an LRT, maximum likelihood (ML) method was used. Accordingly, the model including highest polynomial power over the simplest one was chosen whenever LRT P ≤ 0.100. Next, subjects' group (discharged from ICU and death in ICU) was added as a binary term (0 discharged from ICU, 1 death in ICU). The interaction between group and time (linear, quadratic and cubic) was assessed by LRT and interaction was included in the model if LRT P value ≤ 0.100. Non-significant (P>0.05) fixed effects were removed from the model.

Statistical analyses on time-course of physiological variables during ECMO were performed as previously described (1) and carried out by SAS 9.4 statistical package.

sTable 1. Variables associated with indirect signs of hemolysis during ECMO.

	COHb, mean (mg/dL)			Haptoglobin, mean (mg/dL)		
**Variable**	**Rho**	**95% CI**	**p-value**	**Rho**	**95% CI**	**p-value**
Daily average RBC transfusion, 100 mL	0.442	0.303 – 0.553	*<0.001*	-0.353	-0.497 - -0.184	*<0.001*
Average BF, L/min	-0.052	-0.210 – 0.119	0.529	0.173	-0.005 – 0.348	0.054
Average Pin, mmHg	0.090	-0.075 – 0.267	0.293	-0.146	-0.310 – 0.049	0.104
Drainage cannula size, French	-0.007	-0.174 – 0.162	0.937	0.111	-0.047 – 0.285	0.194
Return cannula size, French	0.001	-0.163 – 0.158	0.989	0.073	-0.088 – 0.236	0.368
ECMO days	0.381	0.232 – 0.531	*<0.001*	-0.273	-0.438 - -0.096	*0.001*
ECMO circuits, n	0.314	0.162 – 0.467	*<0.001*	-0.154	-0.311 – 0.024	0.076
Average circuit duration, days	0.185	0.023 – 0.340	*0.025*	-0.210	-0.368 - -0.045	*0.013*
CRRT during ECMO (Ref. No)	-0.006	-0.163 – 0.172	0.945	-0.157	-0.332 – 0.008	0.068
Average d-Dimer, mg/L	0.184	0.026 – 0.344	*0.021*	-0.041	-0.212 – 0.130	0.637

Abbreviations: COHb=carboxy hemoglobin; CI=confidence interval; RBC=red blood cell; Pin=pressure of the ECMO drainage cannula; BF=blood flow of ECMO pump; ECMO= extracorporeal membrane oxygenation; CRRT=continuous renal replacement therapy.

sTable 2. Unadjusted predictors of VAP by univariate logistic regression analysis

Variable	OR	95% CI	p-value
**Baseline characteristics**			
Year	0.85	0.77-0.93	0.001
BMI	0.91	0.85-0.97	0.008
**Laboratory markers before ECMO cannulation**			
Transferrin before ECMO	0.99	0.98-1.00	0.032
COHb before ECMO cannulation, mg/dL	1.14	1.08-1.21	<0.001
**Indirect signs of hemolysis and hemodynamics, average data during ECMO**			
Mean COHb before VAP, mg/dL	1.01	1.01-1.02	<0.001
**Clinical characteristics before ECMO cannulation**			
Days of mechanical ventilation before ECMO	1.16	1.06-1.27	0.001
**Illness severity before ECMO cannulation**			
Respiratory system compliance at ECMO cannulation	0.93	0.90-0.97	<0.001

sTable 3. Unadjusted predictors of mortality by univariate logistic regression analysis

Variable	OR	95% CI	p-value
**Baseline characteristics**			
Age	1.04	1.01-1.07	0.012
**Clinical characteristics before ECMO cannulation**			
Days of mechanical ventilation before ECMO	1.12	1.04-1.20	0.003
Bacteremia pre-ECMO	3.21	1.04-9.87	0.041
**Illness severity before ECMO cannulation**			
SOFA coagulation	1.54	1.11-2.14	0.010
SOFA total	1.12	1.02-1.23	0.021
Respiratory system compliance at ECMO cannulation	0.96	0.93-0.99	0.009
PRESERVE index	1.58	1.29-1.93	<0.001
RESP index	0.73	0.64-0.85	<0.001
**Laboratory markers before ECMO cannulation**			
Haptoglobin before cannulation	1.00	0.99-1.00	0.009
COHb before cannulation	1.08	1.02-1.13	0.006
**ECMO and laboratory characteristics, average data during ECMO**			
Days to circuit	1.24	1.11-1.39	<0.001
Days of ECMO	1.03	1.01-1.04	0.001
PEEP mean	0.84	0.74-0.95	0.005
Peak pressure	1.13	1.03-1.24	0.010
**Indirect signs of hemolysis and hemodynamics,**			

**average data during ECMO**			
Mean COHb mg/dL	1.01	1.00 – 1.01	0.001
Mean Haptoglobin mg/dL	0.99	0.99-1.00	0.001
Mean mPAP	1.10	1.02-1.18	0.015
Mean BP	0.85	0.79-0.91	<0.001
Mean pulmonary vascular resistance mean	1.01	1.00-1.02	0.002
Mean daily RBC transfusion	1.00	1.00-1.01	0.002
First shunt	1.04	1.01-1.06	0.004
Lactate before ECMO cannulation	1.25	1.06-1.47	0.008
Iron before ECMO cannulation	1.01	1.00-1.02	0.005
**Outcomes**			
ECMO, bacteremia	3.37	1.56-730	0.002
ECMO, VAP	3.41	1.64-7.08	0.001
Aspergillosis	8.06	2.70-24.10	<0.001
CRRT	2.74	1.34-5.58	0.006

sFigure 1. Patient selection criteria. An increasing number of veno-venous ECMO over the years was observed at our center since 2003 until 2019 (i.e. <10, 2003-2008; 10-20 2009-2017; 20-30 in 2018 and 2019).

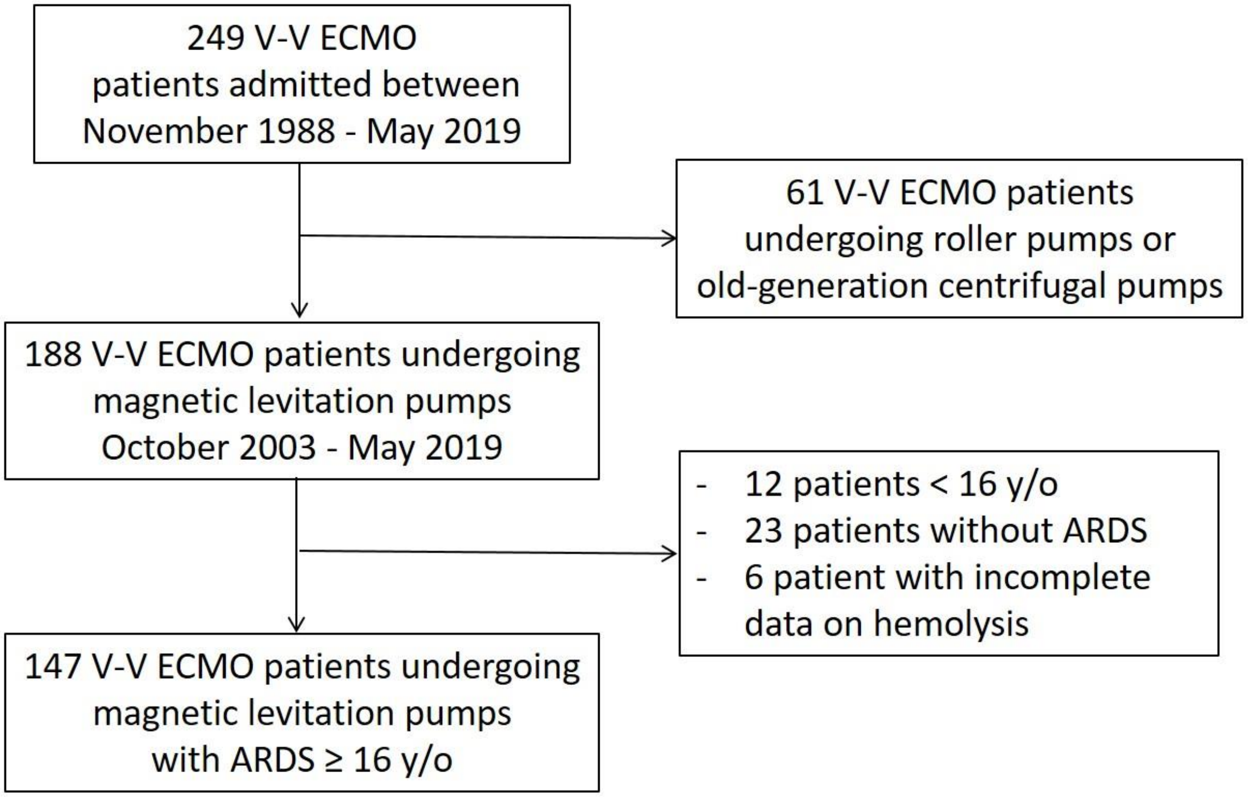

sFigure 2. Correlations between average levels of COHb and Haptoglobin with PVR (**A, B**) and SVR (**C, D**), respectively. Correlations between maximum levels of COHb and minimum levels of Haptoglobin with PVR (**E, F**) and SVR (**G, H**), respectively.

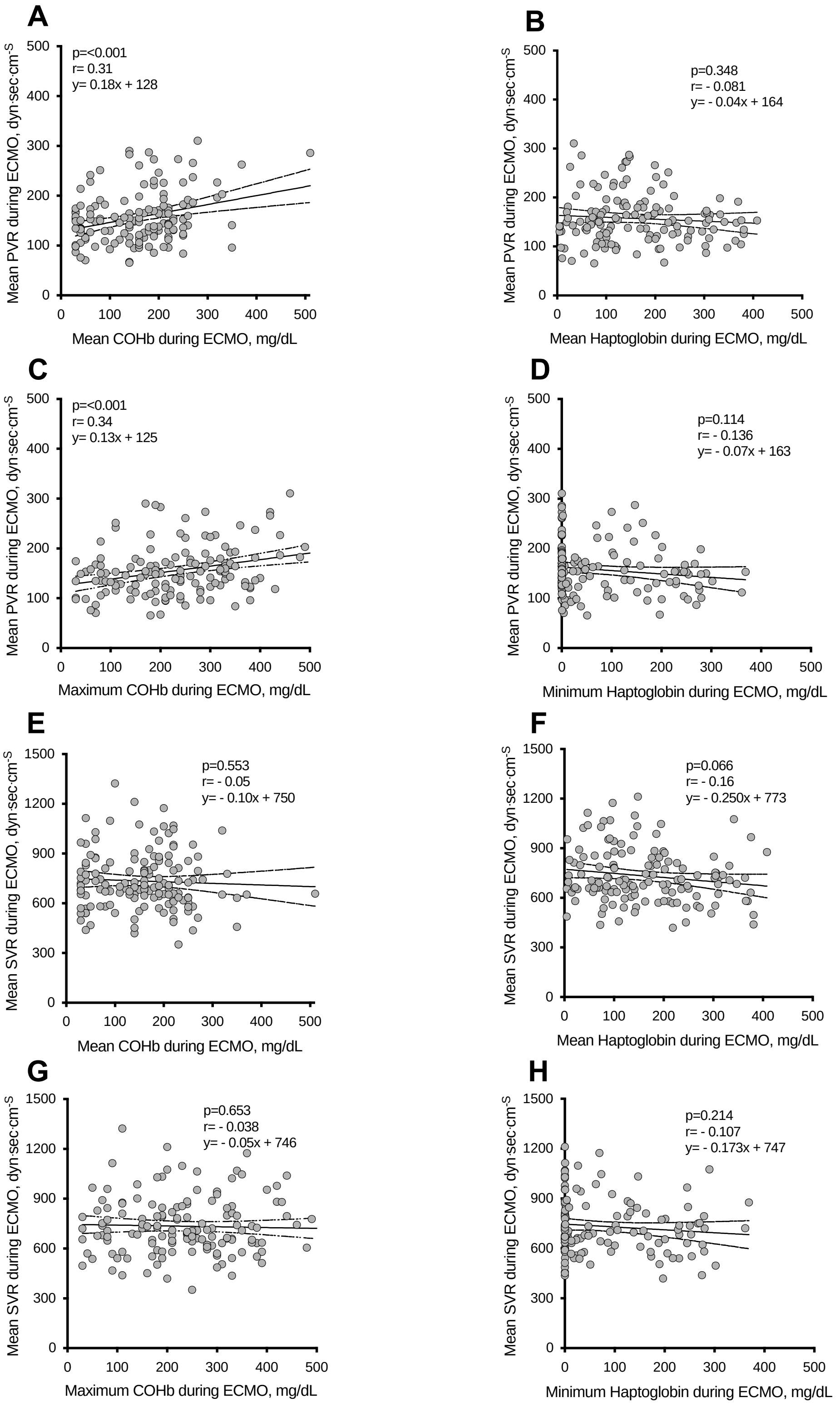

COHb (mg/dL) - Full model parameters – 60 days

Kinetics of COHb (mg/dL) (from ICU admission):

- random intercept at patient level;
- random slope at time level;
- COHb (mg/dL) (dependent variable)
- time as independent variable
- Subject group (death in ICU and discharged from ICU) as a binary term (independent variable)
- Linear, quadratic and cubic term interaction between the 2 independent variables

**Overall model parameters**					
Number of observations **used/** Number of observations **read**	3106/ 3372				
Missing Values (%)	7.9%				
Random intercept group variable: patients	147				
Random slope variable: time					
Observations per patients: (max)	60				
**Model selection**	**P**				
Random effect (assessed on null model)					
Random intercept vs. standard linear regression model	<.0001				
Random slope vs. random intercept model	<.0001				
Functional form of association between time and PF					
Linear vs. quadratic	<.0001				
Quadratic vs. cubic	<.0001				
Interaction between time and group vs. no interaction	<.0001				
**Fixed effect parameters**	**Coeff.**	**std err.**	**95% CI**		**P**
time: linear	5.765	0.571	4.643	6.888	<.0001
time: quadratic	-0.256	0.024	-0.303	-0.209	<.0001
time: cubic	0.003	0.0003	0.002	0.003	<.0001
Group: binary (1 death in ICU , 0 discharged from ICU)	30.654	13.931	3.127	58.182	0.029
Interaction					
time (linear) and group	2.673	0.637	1.397	3.949	<.0001
time (quadratic) and group					removed
time (cubic) and group	-0.0005	0.0001	-0.001	-0.0003	<.0001
Constant: Intercept at time = 0	115.930	8.016	100.100	131.760	<.0001
**Random effect parameters**	**Coeff.**	**std err.**	**95% CI**		**P**
.Random intercept	5674.770	718.090	4267.310	7082.230	<.0001
Unstructured covariance	38.060	22.612	-6.260	82.380	0.092
Random slope	6.780	1.876	3.100	10.460	0.0002

Residual variance	2205.79<br>0	59.216	2089.72<br>0	2321.85<br>0	<.0001

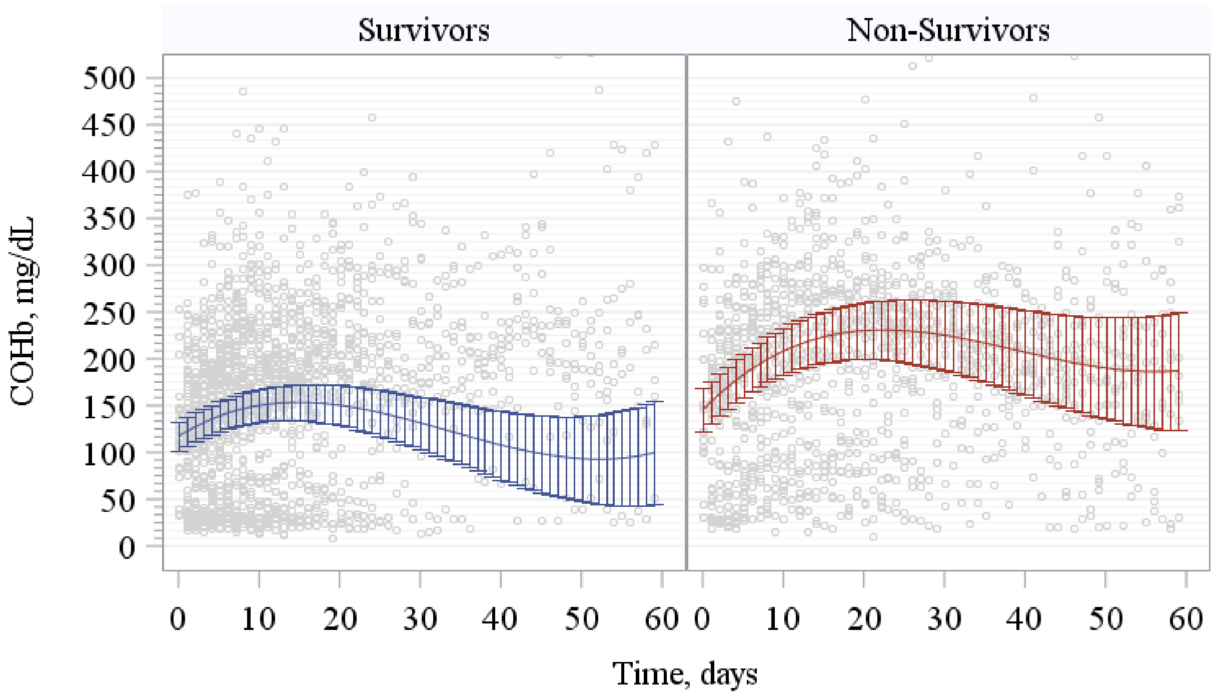

Haptoglobin - Full model parameters – 60 days

Kinetics of Haptoglobin (from ICU admission):

- random intercept at patient level;
- random slope at time level;
- Haptoglobin (dependent variable)
- time as independent variable
- Subject group (death in ICU and discharged from ICU) as a binary term (independent variable)
- No interaction between the 2 independent variables

**Overall model parameters**					
Number of observations **used/**<br>Number of observations **read**	1496/<br>3371				
Missing Values (%)	55.6%				
Random intercept group variable: patients	147				
Random slope variable: time					
Observations per patients: (max)	60				
**Model selection**	**P**				
Random effect (assessed on null model)					
Random intercept vs. standard linear regression model	<.0001				
Random slope vs. random intercept model					
Functional form of association between time and PF					
Linear vs. quadratic	<.0001				
Quadratic vs. cubic	0.0003				
Interaction between time and group vs. no interaction	0.4192				
**Fixed effect parameters**	**Coeff.**	**std err.**	**95% CI**		**P**
time: linear	-11.478	1.010	-13.461	-9.494	<.0001
time: quadratic	0.248	0.045	0.160	0.335	<.0001
time: cubic	-0.002	0.001	-0.003	-0.001	0.0002
Group: binary (1 death in ICU , 0 discharged from ICU)	-63.257	16.489	-95.858	-30.655	0.0002
Interaction					
time (linear) and group					
time (quadratic) and group					
time (cubic) and group					
Constant: Intercept at time = 0	255.890	11.377	233.450	278.330	<.0001
**Random effect parameters**	**Coeff.**	**std err.**	**95% CI**		**P**
Random intercept	11843.000	1571.530	8762.870	14923.270	<.0001

Unstructured covariance	-353.710	82.547	-515.500	-191.920	<.000<br>1
Random slope	29.585	6.787	16.280	42.890	<.000<br>1
Residual variance	3054.830	126.130	2807.61<br>0	3302.050	<.000<br>1

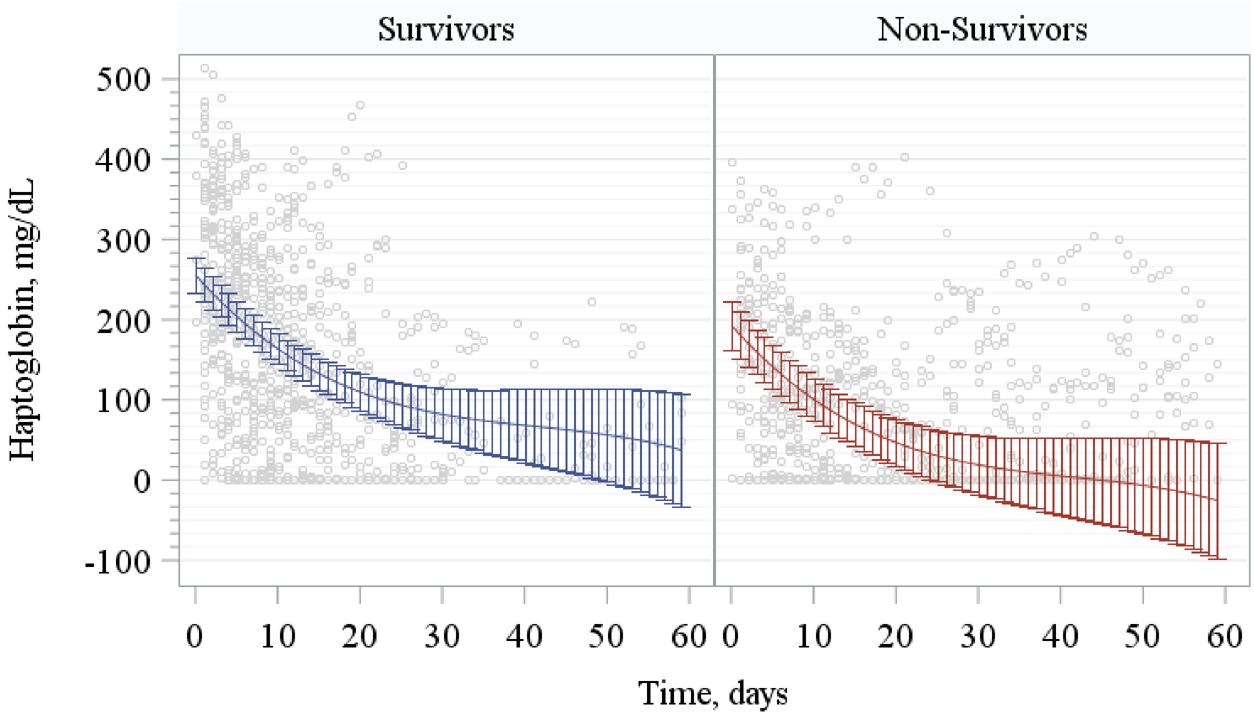

PVR - Full model parameters – 60 days

Kinetics of PVR (from ICU admission):

- random intercept at patient level;
- random slope at time level;
- PVR (dependent variable)
- time as independent variable
- Subject group (death in ICU and discharged from ICU) as a binary term (independent variable)
- Linear and quadratic interaction between the 2 independent variables

**Overall model parameters**					
Number of observations **used/**<br>Number of observations **read**	2579/<br>3372				
Missing Values (%)	23.5%				
Random intercept group variable: patients	147				
Random slope variable: time					
Observations per patients: (max)	58				
**Model selection**	**P**				
Random effect (assessed on null model)					
Random intercept vs. standard linear regression model	<.0001				
Random slope vs. random intercept model	<.0001				
Functional form of association between time and PF					
Linear vs. quadratic	0.0012				
Quadratic vs. cubic	0.5326				
Interaction between time and group vs. no interaction	0.0515				
**Fixed effect parameters**	**Coeff.**	**std err.**	**95% CI**		**P**
time: linear					remove<br>d
time: quadratic					remove<br>d
time: cubic					
Group: binary (1 death in ICU , 0 discharged from ICU)	20.514	9.204	2.340	38.688	0.027
Interaction					
time (linear) and group	1.446	0.518	0.422	2.469	0.006
time (quadratic) and group	-0.027	0.007	-0.041	-0.012	0.0003
time (cubic) and group					
Constant: Intercept at time = 0	147.020	4.693	137.750	156.300	<.0001
**Random effect parameters**	**Coeff.**	**std err.**	**95% CI**		**P**
Random intercept	2370.74<br>0	329.12<br>0	1725.66<br>0	3015.81<br>0	<.0001
Unstructured covariance	-45.649	15.800	-76.620	-14.680	0.004
Random slope	5.248	1.140	3.010	7.480	<.0001

Residual variance	1881.32<br>0	55.210	1773.11<br>0	1989.53<br>0	<.0001

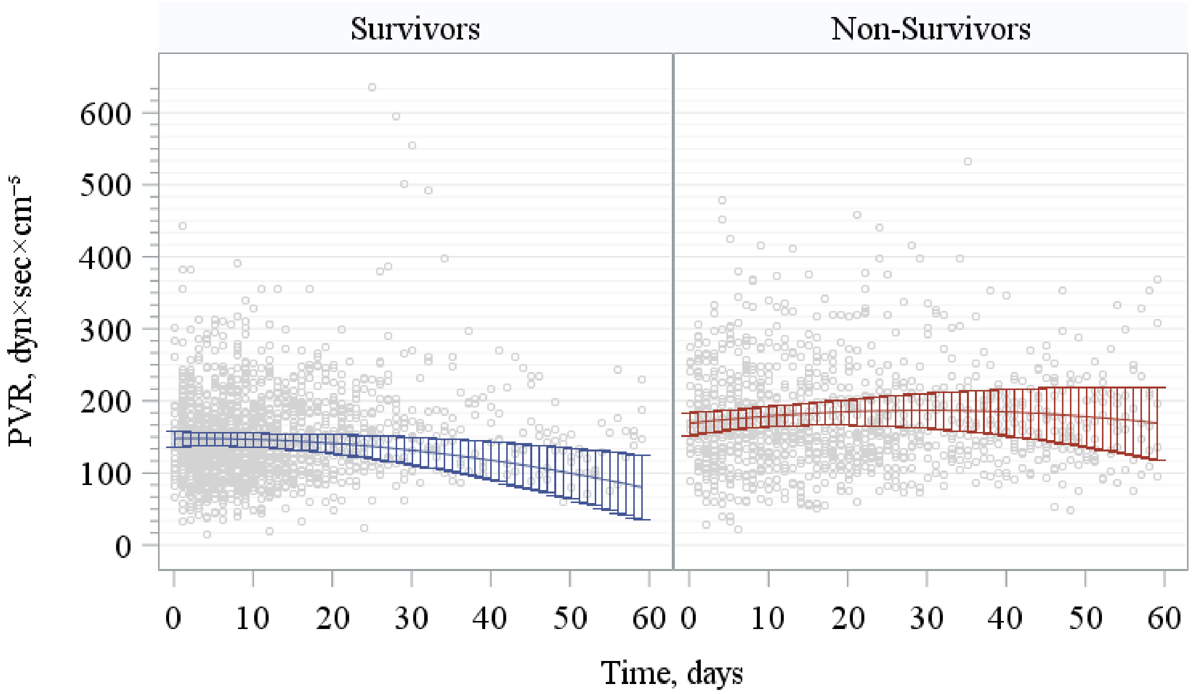

SVR - Full model parameters – 60 days

Kinetics of SVR (from ICU admission):

- random intercept at patient level;
- random slope at time level;
- SVR (dependent variable)
- time as independent variable
- Subject group (death in ICU and discharged from ICU) as a binary term (independent variable)
- Linear, quadratic and cubic interaction between the 2 independent variables

**Overall model parameters**					
Number of observations **used/**<br>Number of observations **read**	3106/<br>3372				
Missing Values (%)	7.9%				
Random intercept group variable: patients	147				
Random slope variable: time					
Observations per patients: (max)	60				
**Model selection**	**P**				
Random effect (assessed on null model)					
Random intercept vs. standard linear regression model	<.0001				
Random slope vs. random intercept model	<.0001				
Functional form of association between time and PF					
Linear vs. quadratic	<.0001				
Quadratic vs. cubic	<.0001				
Interaction between time and group vs. no interaction	<.0001				
**Fixed effect parameters**	**Coeff.**	**std err.**	**95% CI**		**P**
time: linear	-13.346	1.905	-17.086	-9.606	<.0001
time: quadratic	0.695	0.084	0.531	0.860	<.0001
time: cubic	-0.006	0.001	-0.008	-0.004	<.0001
Group: binary (1 death in ICU , 0 discharged from ICU)					remove<br>d
Interaction					
time (linear) and group					remove<br>d
time (quadratic) and group					remove<br>d
time (cubic) and group	-0.002	0.0004	-0.003	-0.001	<.0001
Constant: Intercept at time = 0	778.150	16.886	744.840	811.460	<.0001
**Random effect parameters**	**Coeff.**	**std err.**	**95% CI**		**P**
Random intercept	31015.00<br>0	4321.4<br>0	22545.45<br>0	39485.35<br>0	<.0001
Unstructured covariance	-795.160	240.35<br>0	-1266.240	-324.080	0.001

Random slope	102.180	21.419	60.200	144.160	<.0001
Residual variance	26867.00<br>0	715.84<br>0	25464.06<br>0	28270.16<br>0	<.0001

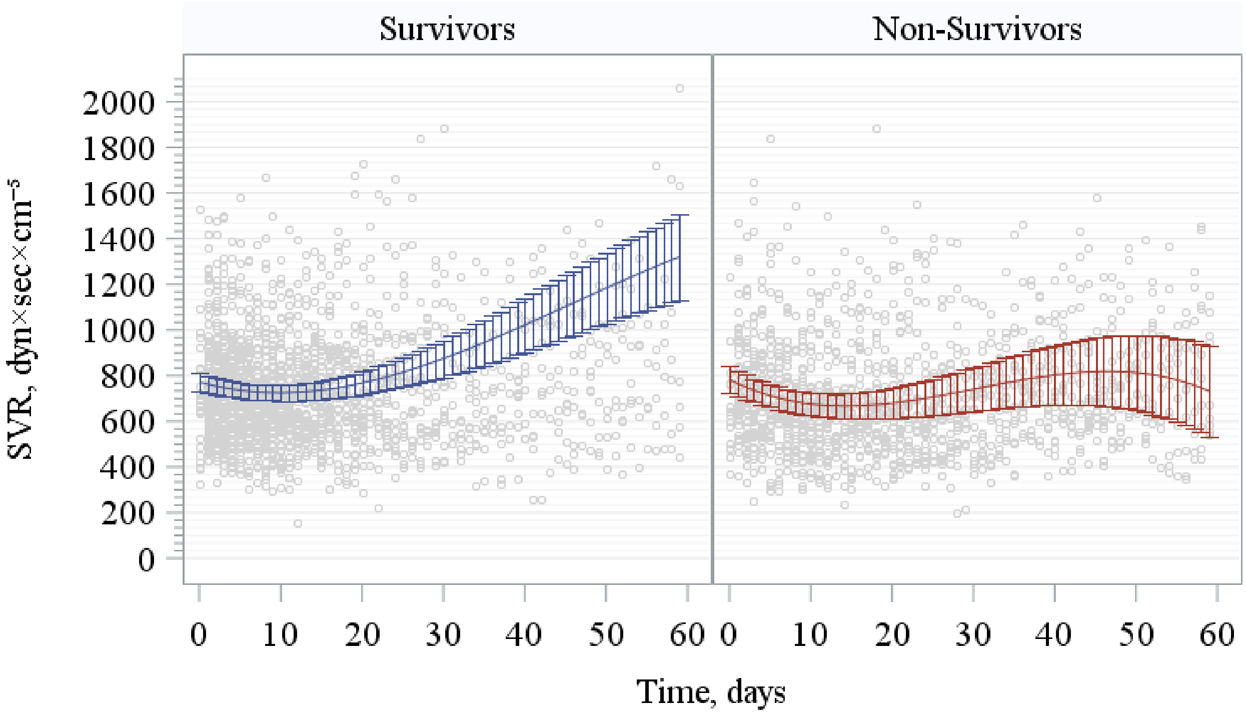

References

1. Lanini S, Portella G, Vairo F, Kobinger GP, Pesenti A, Langer M, Kabia S, Brogiato G, Amone J, Castilletti C, Miccio R, Zumla A, Capobianchi MR, Di Caro A, Strada G, Ippolito G; INMI-EMERGENCY EBOV Sierra Leone Study Group. Blood kinetics of Ebola virus in survivors and nonsurvivors. J Clin Invest. 2015 Dec;125(12):4692-8.
